# Strong Evidences of the Ovarian Carcinoma Risk in Women after IVF Treatment: A Review Article

**Published:** 2019-12

**Authors:** Dariush D. FARHUD, Shaghayegh ZOKAEI, Mohammad KEYKHAEI, Marjan ZARIF YEGANEH

**Affiliations:** 1. School of Public Health, Tehran University of Medical Sciences, Tehran, Iran; 2. Department of Basic Sciences, Iranian Academy of Medical Sciences, Tehran, Iran; 3. School of Advanced Medical Sciences, Islamic Azad University, Tehran Medical Branch, Tehran, Iran; 4. School of Medicine, Tehran University of Medical Sciences, Tehran, Iran; 5. Cellular and Molecular Endocrine Research Center, Research Institute for Endocrine Sciences, Shahid Beheshti University of Medical Sciences, Tehran, Iran

**Keywords:** Ovarian cancer, In-vitro fertilization, Ovarian hyper stimulation, Clomiphene citrate, Gonadotropins, Infertility

## Abstract

**Background:**

In-vitro fertilization (IVF) has been very popular since the birth of the first “test-tube” baby. This method is the last hope and the most serious solution for couples with infertility problems. Although childbearing is a concern of many couples, it must always be noted that any method can also have disadvantages. Thus, many studies have been done on the problems encountered by this method.

**Methods:**

We searched for relevant articles in Pub Med, Springer, Elsevier, and Google Scholar databases, and studied more than 70 papers. Keywords used included ovarian cancer, in vitro fertilization, gonadotropin hormone, clomiphene citrate, and infertility.

**Results:**

Due to the large number of studies, high doses of the ovulation-stimulating drugs and their repeated use in this method can increase the risk of the ovarian hyper stimulation syndrome (OHSS), and ovarian cysts, which can lead to ovarian cancer. Also, an increase in the risk of developing ovarian cancer can be due to the increased duration of treatment for up to 12 months and the high doses of medications that are followed by defecation and failure of this treatment.

**Conclusion:**

Due to the increase in the rates of infertility treatments and the incidence of gynecological cancers, especially ovarian cancer, this method need to be used with caution. IVF clients and candidates should be informed about potential future risks. People should be evaluated genetically for their history of ovarian cancer and be more aware of the importance of “Personalized medicine”.

## Introduction

In-vitro fertilization has been carried out for nearly 40 years, and at the top of the list of countries with optimal performance is Israel, which provided unlimited cycles for this operation up to two children for every women citizen, followed by Denmark, the Netherlands, Sweden, and other countries at the next levels (1). Since the birth of Louise Brown, the first ‘test-tube’ baby, in 1978, in vitro fertilization (IVF) has become a common and popular method of infertility treatment (2, 3) which has steadily risen (4), and maybe the last hope of couples in many countries over the last several decades. However, after discovering a very close relation between doing IVF and congenital malformation and multiple pregnancies, research on the destructive consequences of this method began.

**Fig. 1:**
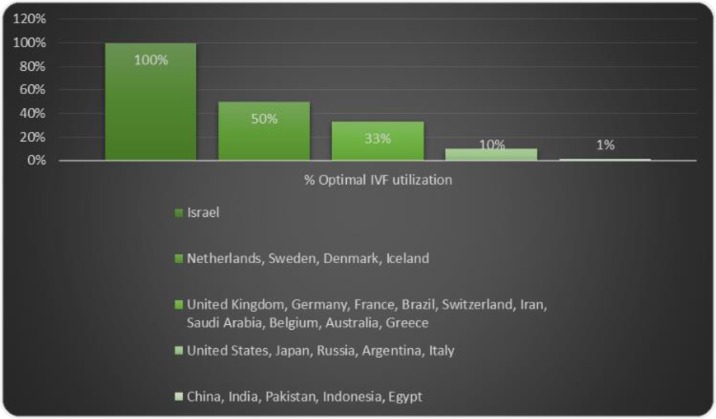
International optimal utilization of IVF (7)

One of the disadvantages of this method is the development of abnormalities which threatens children from IVF (3). But there are many concerns about the health risks for the mothers of offsprings conceived by assisted reproductive technology (ART), of which one of the most serious is ovarian cancer (5). As it is known, there is a close relation between reducing in the risk of female genital cancer and breast cancer, which are hormone-dependent (6), and having at least one child and a history of breastfeeding. Therefore, in this paper, we are considering the risk of ovarian cancer in mothers under IVF who take fertility drugs. (Fig. 1) (7).

Every year, around 239,000 new cases of women in the world are diagnosed with ovarian cancer, with only below 45% survival rates, and according to the death toll (8), it has become the 8th deadly(fatal) cause of cancer death among women (9). It is also diagnosed that serous ovarian tumors can be originated from the fallopian tube instead of the ovary itself (10).

**Fig. 2:**
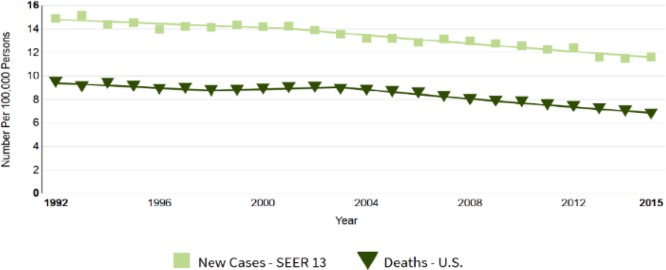
National Institutes of Health (NIH) - National Cancer Institute (12)

Among all gynecological malignancies, ovarian cancer has the worst prognosis, which as a medical term includes several types of tumors with different phenotypes, molecular biology, tumor progression, etiology, and even different prognosis (11). There are many factors which can increase the annual incidence rate of developing ovarian cancer risk such as a family history of the patient or heredity, mutation status, age, number of pregnancies, breastfeeding, physical activity, alcohol consumption and, in general, life style (9) (Fig. 2) (12).

Overall, there is a well-established association with a high proportion of hereditary between ovarian cancer risk and mutations, furthermore, these mutations are also prevalent among patients with ovarian cancer who do not have a family history of ovarian cancer (13). According to the appearance of the epithelium, ovarian tumors are classified into these subtypes: serous, mucinous, clear cell, endometrioid, squamous, transitional, mixed and undifferentiated. These subtypes are also divided into two groups of high-grade and low-grade tumors, based on morphology and genetic alternation.

Low-grade ones, including serous carcinoma, mucinous, endometrioid, and clear cell carcinomas, likely to arise stepwise in an adenoma (borderline tumor) carcinoma sequence from typical to micropapillary borderline tumors to low-grade invasive serous carcinoma, with a lower rate of progression and be caused by mutation in different genes including KRAS, BRAF, PTEN, and beta-catenin, and KRAS or BRAF mutations lead to the effective activation of the MAPK signaling in low-grade serous carcinoma cells (14). Contrary to the previous type, the high-grade type consists of the high-grade serous carcinoma, malignant mixed mesodermal tumors (carcinosarcomas) and undifferentiated carcinomas, grows rapidly and aggressively, with a high level of genetic stability characterized by TP53 mutations and BRCA1 and BRCA2 dysfunction (15, 16) (Table 1) (14).

**Table 1:** Mutations, Precursors, Chromosomal Instability of Type I and Type II Carcinomas (14)

***Type I (Low-grade)***	***Mutations***	***Precursors***
Low-grade endometrioid CA	CTNNB1, PTEN	Endometriosis
Low-grade serous CA^b^	KRAS, BRAF	Serous borderline tumor
Mucinous CA	KRAS	Mucinous borderline tumor
Most clear cell CA^c^	PIK3CA	Endometriosis
Type II (High-grade)	Mutations	Precursors
Carcinosarcoma, High-grade serous CA, endometrioid CA	TP53	Not recognized
Clear cell CA^c^, Undifferentiated CA	__	

Although high-grade cancers arise without an easily identifiable precursor lesion, most low-grade cancers arise from cystadenomas or endometriosis (16). Most ovarian cysts or benign ovarian tumors will never develop ovarian cancer. But some of these cysts or benign tumors, produced by the low-grade pathway, have the potential for malignancy and can become cancerous, like mucinous and endometrioid tumors. So eliminating these benign cysts may reduce the mortality rate of ovarian cancer (17–21).

**Fig. 3:**
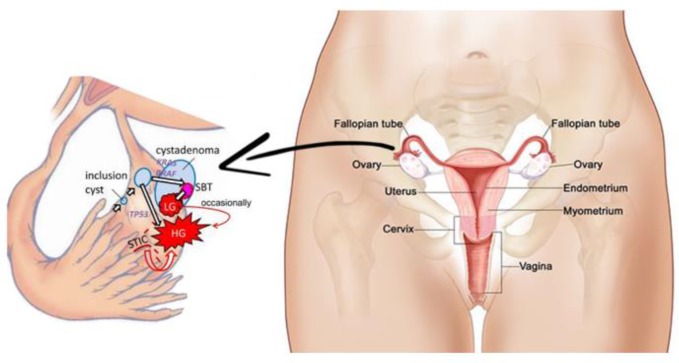
Low-grade (arose from a serous cystadenoma) and high-grade (arose from a serous tubal intraepithelial carcinoma) serous carcinoma. Respectively, due to mutations in the KRAS / BRAF / ERRB2 or TP53, low-grade or high-grade carcinoma are developed (10, 12)

**Fig. 4:**
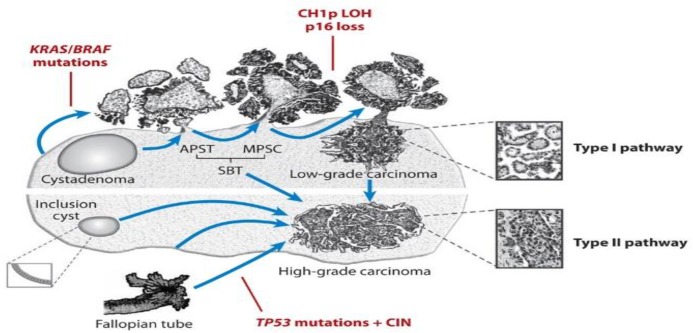
High-grade and low-grade ovarian serous carcinoma pathogenesis. There are two different pathways of ovarian cancer. Low-grade carcinoma, which has a lower rate of progression than high-grade carcinoma, is caused by serous cystadenomas with mutations in KRAS and BRAF. High-grade carcinoma with TP53 mutation has a higher rate of progression and can also spread to the pelvic and peritoneal organs. The precursor lesions of this high-grade carcinoma is also not well known, but it can originate from the epithelial inclusion of the ovary or the distal of the fallopian tube (14)

Overall, the aim of our study was to evaluate the possible effects of ovulation stimulants, used in fertility processes, on the increased risk of developing cysts and benign tumors that could lead to ovarian cancer. Therefore, we briefly discuss the mechanism of these stimulant drugs. (Fig. 3) (10, 12), (Fig. 4) (14).

### Effect of Fertility Drugs and Medications

Infertile women are more at risk for the development of ovarian cancer (22), so that parous women compared to nulliparous women had an estimated 26% lower risk of ovarian cancer (23), but in some available studies, especially those with great sample size, have been referred to the effects of hormonal drugs used in infertility treatment on the development of ovarian cancer (24), as in a study of 255,786 women in UK, with an average of 8.8 years’ follow-up, 386 cases of ovarian cancer have been reported. Women who had no live births by the end of treatment, were at greatest increased risk of ovarian cancer due to the high doses of these medications at each stage of the IVF (25). In recent decades, increasing infertility has also increased the use of ovulation-stimulating drugs, gonadotropin, and repeated ovulation hormones (26, 27), and fertility medications, which has raised concerns about their possible long-term carcinogenic effects, especially on the ovary (28, 29), because of the promoting polyfollicular ovulation (30). Thus, there should be an association among drug type, dosage, duration, and waiting more than 1 year to conceive a baby (31).

Here is a theory for the pathogenesis of ovarian cancer caused by fertility drugs that repeated ovulations by overstimulation of the ovary disrupts the ovarian epithelium and leads to the malignant transformation of ovarian epithelial cells (32). For instance, there was an increase in the incidence of ovarian cancer in women treated with tamoxifen or clomiphene citrate (CC) (33), while some studies with small sample size were not statistically significant (34).

### Clomiphene Citrate (CC)

CC, a nonsteroidal ovarian stimulant, used alone or in combination with intrauterine insemination (IUI) to induce a multifollicular response, and thus increase the risk of conceiving twins, and may increase the risk of ovarian cancer and obesity (35). Clomiphene compound consists of both cis and trans isomers, which the isomer designated by the manufacturers as the cis compound is more effective in stimulating ovulation (36, 37). CC function is to connect to hypothalamic nuclear estrogen receptors, which by negative feedback causes the secretion of GnRH, increases gonadotrophin secretion and ovarian follicular activity (38–40). Although in another study conducted by Smith and her colleagues, they pointed out that clomiphene affects directly on the ovaries to stimulate biosynthesis of estrogen and raise estrogen levels, which consequently, stimulate gonadotrophin release by the pituitary (41–44).

Treatment with CC increases the pulse amplitude (not the frequency), because in infertility due to polycystic ovary syndrome (PCOS), the GnRH pulse frequency is abnormally high (45). In anovulatory cycles, serum LH, FSH, and Estradiol (E2) increased initially, then, unlike the ovulatory cycles, returns to baseline and remained unchanged for the ensuing 40 days.

But on contrary, ovulation induction due to the use of clomiphene citrate in women with PCOS is associated with increased secretion of LH and FSH with enhanced estrogen secretion. Also, it is evident that clomiphene can affect pituitary function (46). Therefore, increasing the LH pulse amplitude after CC, along with the reduction of the pituitary sensitivity to GnRH, indicates a hypothalamic effect (45), although studies show that the minimum effective dose of CC in inhibiting estradiol absorption by the anterior hypothalamus was 100 times higher than the anterior pituitary, which is more sensitive than the former to CC (46). CC remains attached to the nuclear ER for a longer period of time than estrogen, and by intervening in the normal process of ER replenishment, reduces the concentration of ER (47).

One of the side effects of using CC is ovarian cysts formation. With decreasing dosage or duration of treatment, the emergence of ovarian cysts has also declined (39). Thus, studies show an increased risk of ovarian cancer in both women with or without ovulatory abnormalities, who used CC for a long-term (48).

### Tamoxifen

Tamoxifen is a selective estrogen receptor modulator (SERM) which can bind to the receptors due to structural similarity to estrogen. Antiestrogens have had their greatest clinical usefulness as inducers of follicular maturation in women with fertility problems (amenorrheic or oligomenorrheic women), although initially were considered as antifertility agents (49, 50). This anti-estrogenic compound may be used as a substitute for clomiphene citrate with a similar function to induce ovulation in women with anovulatory infertility (51) due to Polycystic Ovarian Syndrome (PCOS). The early follicular phase administration of tamoxifen increased the serum concentration of estradiol (E2) during the follicular phase, at the mid-cycle, and at the times of mid-luteal hormone peaks, and was accompanied by a significant increase in integrated luteal phase progesterone (P) concentration (38, 52, 53).

### Gonadotropins

Gonadotropins are other agents for ovarian stimulation and ovulation induction in women undergoing assisted reproductive technology (ART) or women with anovulatory problems (38). Gonadotropins play an important role in the development of ovarian cancer, approximately, at a rate of 40% in epithelial ovarian cancer (54). During IVF, high doses of gonadotropin may be used as a result of several injections per day. The most effective form of treatment for subfertility is intrauterine insemination combined with ovarian hyperstimulation, and gonadotropins might be those effective agents (55), but with low dose protocols to reduce the risk of multiple pregnancies and risks of ovarian hyperstimulation syndrome (56). In the first step of IVF, hormonal drugs are used to stimulate the ovaries. Muscular injection of human menopausal gonadotropins (HMG), found in the urine of postmenopausal women, stimulates the ovaries to grow follicles (57). Human Chorionic Gonadotropin (HCG), obtained from the urine of pregnant women, is also used for ovulation induction, which induces final follicular maturation and oocyte retrieval in anovulatory patients undergoing ART (58). It also initiates rupture of the preovulatory ovarian follicle according to its activities as an analog of LH (38). As already mentioned, one of the complications of using ovulation induction drugs is ovarian cysts that have been observed in the series of gonadotropin drugs, and approximately 20% of patients receiving GnRH during the follicular or luteal phase had developed ovarian cysts, so that the number of cysts in the follicular phase was higher than the luteal phase, and compared to the follicular phase cysts, the luteal phase cysts are more benign (59). (Table 2) (54, 60–70).

**Table 2:** Function, side effects and potential risks of clomiphene citrate, tamoxifen, HMG, and HCG

***Drugs***	***Function***	***Side effect***	***Potential risks***	***Ref .Nr***
Clomiphene citrate	Non-steroidal ovulation stimulant, an estrogen agonist or antagonist,	Abnormal vaginal/uterine bleeding, vaginal dryness, breast tenderness or discomfort, ovarian enlargement, vomiting, diarrhea, nausea, headache, blurred vision or other visual disturbances, stroke or chest pain, weight gain	Twin or multiple pregnancy, ovarian hyper stimulation syndrome (OHSS), ovarian cysts, ovarian cancer	(60–63)
Tamoxifen	An anti-estrogen in the mammary tissue, also a selective estrogen receptor modulators	Abnormal vaginal bleeding, pain or pressure in the pelvis, leg swelling or tenderness, shortness of breath, weakness, tingling, vision problems, severe headache, blood clots, stroke, hot flashes, nausea, fatigue, mood swings, depression, hair thinning, dry skin, loss of libido	Ovarian cancer, endometrial cancer	(64–66)
HMG (human menopausal gonadotropins)	A mixture of FSH and LH, used to stimulate ovulation	Ovarian enlargement and discomfort, stomach pain, mood swings, fever, headaches, breathing trouble, bloating, skin rash, allergic reactions	Ovarian cancer, ovarian cysts, ovarian hyper stimulation syndrome, multiple pregnancy, ectopic pregnancy	(67–69)
HCG (Human Chorionic Gonadotropin)	Used to induce final maturation of follicle and subsequent ovulation and luteal phase support	Ovarian enlargement, rise in basal body temperature, abdominal bloating and discomfort, pelvic pain, lower abdominal pain, nausea, vomiting	Ovarian cancer, ovarian cysts, ovarian Hyper stimulation Syndrome, multiple pregnancy, ectopic pregnancy	(54, 70)

## Conclusion

IVF, which has been highly regarded as a method of treatment for infertility, can carry risks like any other method. Studies in this field give us different results so that in some studies with small sample size, no significant results have been achieved. However, other studies with a large sample size in this field clearly show the risk of developing ovarian cysts and ovarian cancer. The drugs used in this method, like clomiphene citrate and gonadotropins, extremely hyper-stimulate the ovary, leading to twin or multiple pregnancies, increased ovarian cyst and risk of ovarian cancer. The failure of each cycle compels the couples to try subsequent cycles, in which the dose and duration of the drug intake are increased. Altogether, different aspects of IVF courses should be considered. Initially, the couple should be completely aware of the risks associated with this treatment. Each couple should enter these therapies with regard to their “personalized medicine” in order to avoid long-term infertility treatment in the event of inherited risk of ovarian cancer. Every patient, especially susceptible one, should be monitored closely by the doctor and appropriate tests.

## Ethical considerations

Ethical issues (Including plagiarism, informed consent, misconduct, data fabrication and/or falsification, double publication and/or submission, redundancy, etc.) have been completely observed by the authors.
